# Biological response of zebrafish embryos after short-term exposure to thifluzamide

**DOI:** 10.1038/srep38485

**Published:** 2016-12-07

**Authors:** Yang Yang, Wenxian Liu, Xiyan Mu, Suzhen Qi, Bin Fu, Chengju Wang

**Affiliations:** 1 College of Sciences, China Agricultural University, Beijing, People’s Republic of China; 2 State Key Laboratory of Grassland Agro-ecosystems, College of Pastoral Agriculture Science and Technology, Lanzhou University, Lanzhou, People’s Republic of China; 3 Center of Fishery Resources and Ecology Environment Research, Chinese Academy of Fishery Sciences, Beijing, People’s Republic of China

## Abstract

Thifluzamide is a new amide fungicide, and its extensive application may have toxic effects on zebrafish. To better understand the underlying mechanism, we investigated in detail the potential toxic effects of thifluzamide on zebrafish embryos. In the present study, embryos were exposed to 0, 0.19, 1.90, and 2.85 mg/L thifluzamide for 4 days. Obvious pathological changes were found upon a histological exam, and negative changes in mitochondrial structure were observed under Transmission Electron Microscopy (TEM), which qualitatively noted the toxic effects of thifluzamide on embryos. Moreover, we quantitatively evaluated the enzyme activities [succinate dehydrogenase (SDH), superoxide dismutase (SOD), catalase (CAT), glutathione peroxidase (GPx), caspases], the contents of malonaldehyde (MDA) and interleukin-8 (IL-8) and the expression levels of the related genes. This study suggests that the negative changes in mitochondrial structure and SDH activity might be responsible for oxidative damage, cell apoptosis and inflammation, which would facilitate the action of these factors in cell death and might play a crucial role during toxic events. In addition to providing the first description of the mechanism of the toxic effects of thifluzamide on embryos, this study also represents a step towards using embryos to assess mitochondrial metabolism and disease.

As a thiazole carboxanilide fungicide, thifluzamide controls an extensive range of basidiomycete diseases by inhibiting succinic dehydrogenase (SDH) in the tricarboxylic acid cycle of fungi^1^,^2^. Due to its non-volatility, high potential leachability and abuse, thifluzamide residue was inadvertently released into the environment. Regarding its transfer into aquatic environments, more consideration should be given to the potential toxic effects of thifluzamide on aquatic organisms. Some reports have shown that thifluzamide was toxic to fish and other aquatic invertebrates^3^. Our previous study found that thifluzamide was toxic to three stages of zebrafish (adult, larvae, and embryo) and induced death and malformation^4^. However, limited studies have been conducted on the negative effects of thifluzamide exposure on fish, especially in the early life stages. Thus, it is necessary to perform environmental toxicological studies of thifluzamide to understand its environmental risk during the early life stages of fish.

As fish are directly exposed to contaminants through surface run-off or indirectly exposed through the ecosystem food chain; thus, they are considered bio-indicators of environmental pollution and play increasingly important roles in evaluating the potential environmental risk and aquatic toxicity of chemicals^5^. In recent years, zebrafish as typical small tropical aquarium fish have been used for acute and chronic tests, particularly in the fields of developmental biology and genetics^6^,^7^. Considering the characteristic of embryos, such as external fertilization, rapid development, small size, transparency, and easy maintenance and handling, performing the toxicity test on embryos instead of adult fish has caused concerns for ecological safety. The morphological toxicity of embryos when exposed to chemicals during development is easy to understand, and experiments on embryos are more ethical and economical than those on adult fish^8^,^9^. In addition, many reports have shown that embryos are more sensitive to chemicals than are adult fish^10^,^11^,^12^. More importantly, our previous study indicated that thifluzamide was more toxic to embryos in an acute test^4^. Embryos undergo anatomical and physical changes, and their detoxification mechanisms are not completely developed yet^13^,^14^; thus, toxic effect might be rapid. Embryos complete their development into juveniles in approximately 26 days^15^. These facts might result in the increased sensitivity of embryos exposed to chemicals, but the mechanism has not been determined. Further studies on the toxic effects of thifluzamide on embryos are necessary to determine its safe use in the ecosystem.

In the present study, embryos were used to investigate the developmental toxicity induced by thifluzamide and its related mechanisms. Histopathology and Transmission Electron Microscopy (TEM) were used to measure the morphology of embryos and the structure and ultrastructure of cells to qualitatively evaluate the developmental toxicity. Measurements of the enzyme activities [SDH, superoxide dismutase (SOD), catalase (CAT), peroxidase (GPx), caspases], the malonaldehyde (MDA) and interleukin-8 (IL-8) contents, and the related gene expression levels were used to quantitatively assess mitochondrial damage, oxidative stress, cellular apoptosis and immune response to comprehensively explore the toxicity mechanism. In addition, the present study aimed to provide new insight into the application of embryos to assess mitochondrial diseases.

## Results

### Solvent effect and chemical analysis

The deviations between the theoretical and actual concentrations were less than 20% (Table 1). All of the test solutions in this study were renewed daily. Therefore, the theoretical dosage represented the actual content in this experiment^16^. Data analyses indicated no obvious difference between the solvent control and blank control for all indexes in this study; thus, the test data of the blank control could be used for the following discussion.

### Histopathological changes in embryo

**Figure 1:**
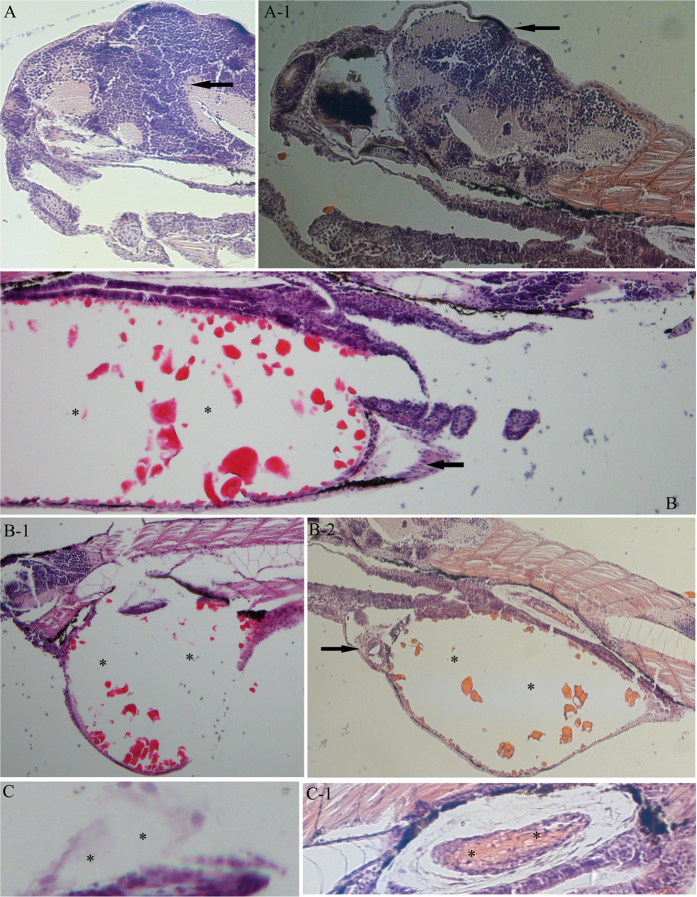
Histologic lesions in zebrafish embryos after exposure to thifluzamide for 4 days. (**A**) Brain from control group (arrows) (200×); A-1. Brain from 2.85 mg/L thifluzamide group showing obvious decreased cells number (arrows) (200×); (**B**) Yolk sac (asterisks) and pericardial region (arrows) from control group (100×); B-1. Yolk sac edema caused by 2.85 mg/L thifluzamide (asterisks) (100×); B-2. Expansion of pericardial space (arrows) due to pericardial edema in larvae (100×) and yolk sac edema (asterisks) caused by 2.85 mg/L thifluzamide (100×); (**C**) Gas bladder from control group showing inflated lumen (asterisks) (100×); C-1. Uninflated gas bladder caused by 2.85 mg/L (asterisks) (100×).

**Figure 2:**
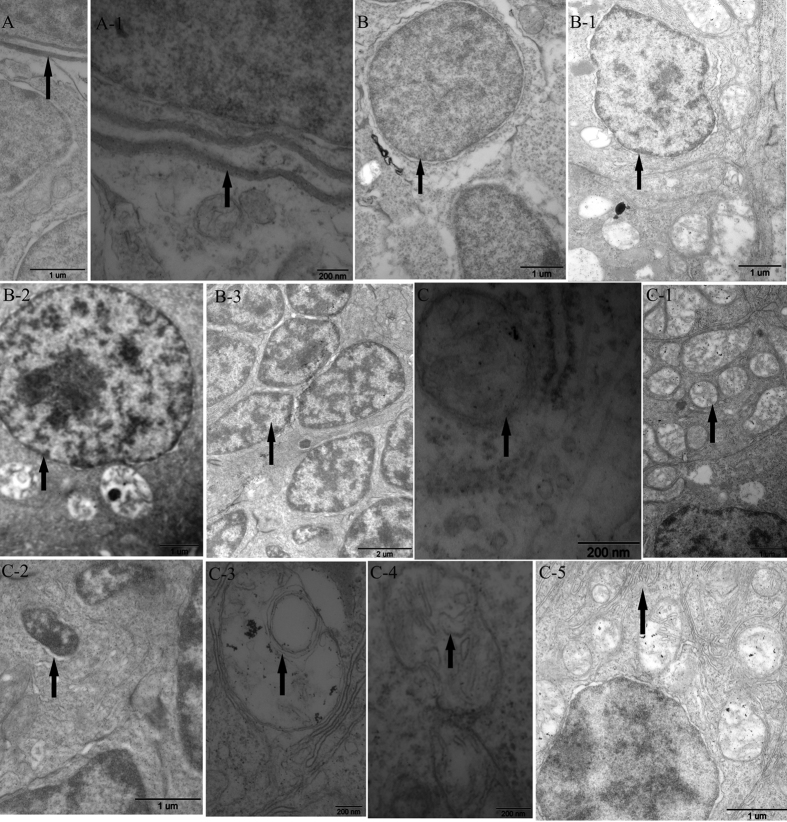
TEM observation in embryos after exposure to thifluzamide for 4 days. (**A**) Intact cell membranes from control group; A-1. Cell membranes from 0.19 mg/L groups; (**B**) Large, round nucleus from control groups; B-1. Nuclei deformation from 0.19 mg/L groups; B-2. Chromatin condensation from 0.19 mg/L groups; B-3. Chromatin condensation and nuclear fragmentation from 2.85 mg/L groups; (**C**) Mitochondria from control group showing distinct crista and intact membranes; C-1. Mild damage to mitochondria from 0.19 mg/L groups; C-2. Apoptotic body from 0.19 mg/L groups; C-3. Mitochondrial damage showing crista degradation from 2.85 mg/L groups; C-4. Mitochondrial swelling showing membrane degradation from 2.85 mg/L groups; C-5. Endoplasmic reticulum breakup from 2.85 mg/L groups. Arrows indicate the different sections.

Histopathology results are presented in Fig. 1 and Table 2. Cells resembling lymphocytes were widely observed in the head and the end of the tail and contained a large, round, and hyperchromatic nucleus (Fig. 1A). Some signs of pathological changes were observed in the treatment groups, such as fewer cells (Fig. 1A-1), yolk sac edema (Figs 1B-1, B-2), pericardial edema (Fig. 1B-2), and uninflated gas bladder (Fig. 1C-1). Lesions were concentration dependent and most marked in the 1.90 and 2.85 mg/L groups. Yolk sac edema was found in all treatment groups, and the rate of occurrence increased in a concentration-dependent manner. Pericardial edema was observed at 1.90 and 2.85 mg/L and usually occurred with yolk sac edema. Uninflated gas bladder and severe brain damage also presented at the two higher concentrations.

### TEM observation

**Figure 3:**
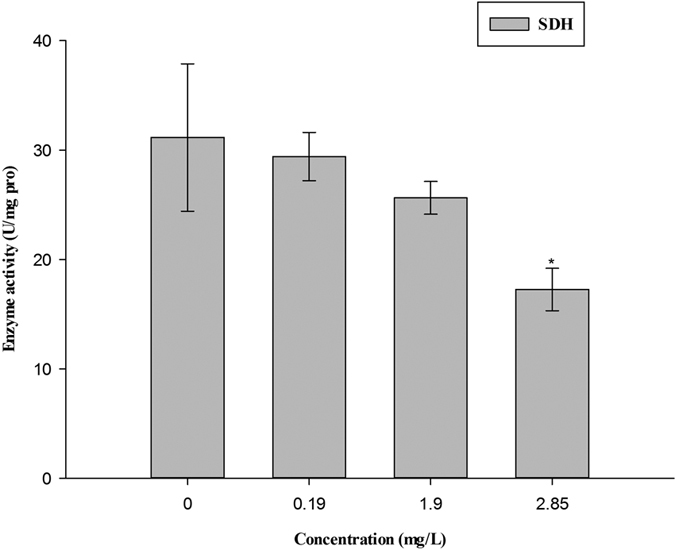
The SDH activity in zebrafish embryos after exposure to thifluzamide for 4 days. Asterisks indicate significant differences between treatments and control. Error bars indicate standard deviation.

**Figure 4:**
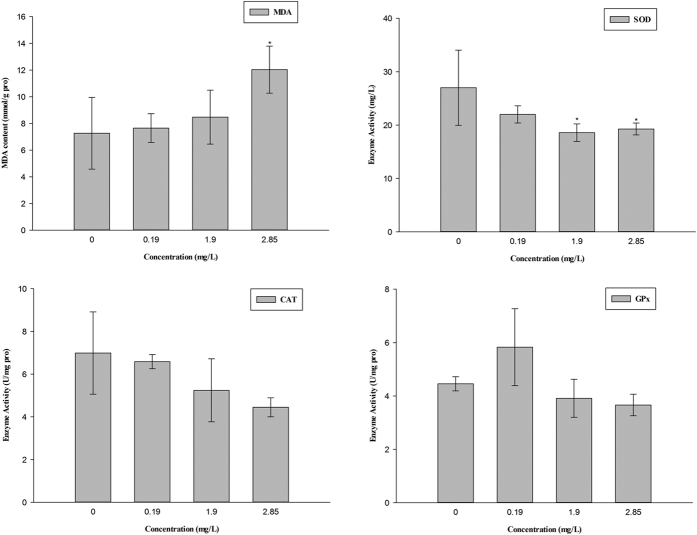
The enzyme activities of SOD, CAT and GPx and the content of MDA in zebrafish embryos after exposure to thifluzamide for 4 days. Asterisks indicate significant differences between treatments and control. Error bars indicate standard deviation.

**Figure 5:**
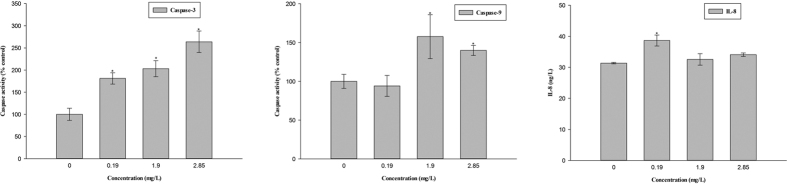
Caspase-3 and Caspase-9 activities and IL-8 content in zebrafish embryos after exposure to thifluzamide for 4 days. Asterisks indicate significant differences between treatments and control. Error bars indicate standard deviation.

To accurately evaluate cellular and subcellular structures, embryos in the various dosage groups were sampled for TEM observation. At 0.19 mg/L, cells presented apoptotic features, such as an intact cell membrane (Fig. 2A-1), some cell nuclei with an irregular shape (Fig. 2B-1), chromatic agglutination (Fig. 2B-2), and apoptotic body formation (Fig. 2C-2). Ultra-microscopic structural analysis showed that the intracytoplasmic organelles, especially mitochondria, significantly increased in number, and the mitochondria showed mild lesions (Fig. 2C-1). With increased concentrations, the number of damaged cells and degree of damage increased. At 1.9 and 2.85 mg/L, the cell damage was more severe, with condensed chromatin and degraded nucleoli (Fig. 2B-3). Meanwhile, intracytoplasmic organelles decreased in number and were obviously damaged. The mitochondria were swollen, bubbled and deformed; in addition, the outer membrane and cristae were even blurred and partly disappeared (Figs 2C-3,C-4). The endoplasmic reticulum (ER) was fractured (Fig. 2C-5).

### Measurement of enzyme activities and MDA content

The activity of SDH was significantly inhibited at 2.85 mg/L thifluzamide (Fig. 3). The activity of SOD obviously decreased at 1.90 and 2.85 mg/L. The activities of GPx and CAT did not show any appreciable changes in the treatment groups. However, the GPx and CAT activities were concentration dependent. The MDA content increased with increasing concentrations and showed significant increase at 2.85 mg/L (Fig. 4).

### Caspase activity and IL-8 content

The activity of Caspase-3 obviously increased at 0.19 mg/L or higher compared with the controls. Caspase-9 activity significantly increased at 1.90 and 2.85 mg/L, but slightly decreased at 0.19 mg/L (Fig. 5).

The content of IL-8 was higher in all treatment groups compared with the controls. In particular, the IL-8 content significantly increased at 0.19 mg/L (Fig. 5).

### Gene expression

**Figure 6:**
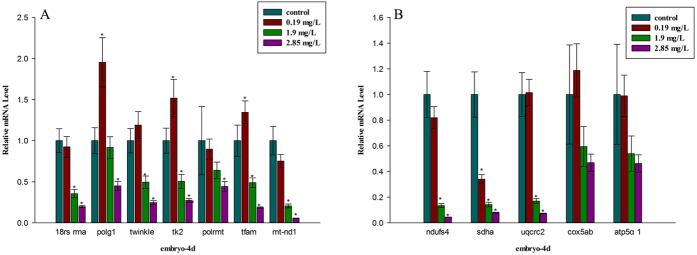
The expression level of genes related to mtDNA replication and transcription and genes encoding components of oxidative phosphorylation (OxPhos) complexes in zebrafish embryos after exposure to thifluzamide for 4 days. (**A**) Expression of mtDNA replication and transcription related genes; (**B**) expression of mitochondrial complexes related genes. Asterisks indicate significant differences between treatments and control. Error bars indicate standard deviation.

The expression levels of a set of genes [18 S small subunit ribosomal RNA (*18rs rrn*), mtDNA polymerase γ (*polg1*), deoxyribonucleoside kinase (*tk2*), mtDNA helicase (*twinkle*), mitochondrial DNA (mtDNA)-directed RNA polymerase (*polrmt*), mitochondrial transcription factor A (*tfam*) and NADH–ubiquinone oxidoreductase subunit 1 (*mt-nd1*) related to mtDNA replication and transcription] were significantly inhibited by 1.90 and 2.85 mg/L thifluzamide (Fig. 6A). In addition, the expression levels of some nuclear-encoded subunits of the mitochondrial respiratory complexes [NADH–ubiquinone oxidoreductase iron–sulfur protein 4 subunit (*ndufs4*), succinate dehydrogenase complex subunit A (*shda*), ubiquinol–cytochrome c reductase core protein II (*uqcrc2*)] were also obviously inhibited by 1.90 and 2.85 mg/L thifluzamide. However the expression levels of cytochrome c oxidase subunit Va (*cox5ab*) and TP synthase mitochondrial F1 complex alpha subunit (*atp5α1*) did not show any appreciable changes in the treatment groups (Fig. 6B). In particular, the *shda* transcription level obviously decreased at 0.19 mg/L.

**Figure 7:**
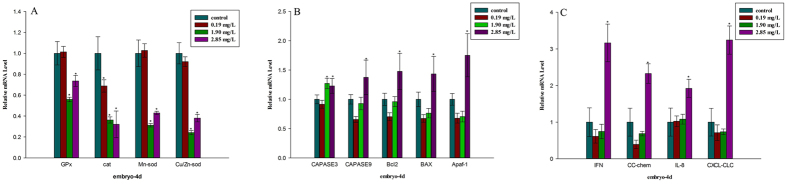
Expression of genes related to oxidative stress, apoptosis and immunity in zebrafish embryos after exposure to thifluzamide for 4 days. (**A**) Expression of oxidative stress-related genes; (**B**) Expression of apoptosis-related genes; (**C**) Expression of immune-related genes. Asterisks indicate significant differences between treatments and control. Error bars indicate standard deviation.

For the genes related to oxidative stress, the transcription of *Cu/Zn–Sod, Mn-Sod*, and *GPx* was not obviously altered by 0.19 mg/L thifluzamide compared to the controls, except for *Cat*, which showed a significant decrease at the transcript level. However, all of the genes related to oxidative stress were significantly down-regulated at 1.90 and 2.85 mg/L (Fig. 7A). The expression of all of the apoptosis-related genes was significantly up-related at 2.85 mg/L (Fig. 7B). The expression of genes related to the immune system significantly increased at 2.85 mg/L. At 0.19 and 1.90 mg/L, no obvious regulation was observed in the embryonic transcription of all immune-related genes (Fig. 7C).

## Discussion

The increasing awareness of a need for pesticides evaluations is well recognized by researchers and practitioners undertaking them. The methods used by experts in conducting toxic evaluations and exploring the toxicity mechanism are many and varied and reflect the particular question or hypothesis being examined. The lethality result indicated that embryos were more sensitive to thifluzamide than adult zebrafish^4^. This study evaluated the toxicity of thifluzamide on the embryos and further explored the toxicity mechanism.

A previous study showed that thifluzamide is lethal and developmentally toxic to zebrafish embryos by quantitatively analysing the mortality, hatching rate, heartbeat rate, etc^4^. Embryos were examined for the presence of pathological changes according to the OECD guidelines^17^. Histopathology can evaluate the toxicity of chemicals on organization. Some studies have used this index to compare the tissue-toxic effects induced by a set of reference compounds^18^,^19^. In the present study, a histological exam qualitatively showed structural alterations in organogenesis, such as yolk sac edema, pericardial edema and uninflated gas bladder. This suggested that the development of embryos was obviously inhibited, which was consistent with the previous study^4^.

In addition, mitochondria play fundamental roles in both life and death, providing ATP and serving as gates of apoptosis for cells^20^. There is no exception in embryonic development. Mitochondria provide energy to embryos from the beginning of fertilization to ensure that the development of an embryo is clearly documented^21^,^22^. Mitochondrial damage and energy metabolism disorder would cause diseases^23^,^24^. Ultrastructural exam showed that the morphology and structure of mitochondria were damaged by thifluzamide. In particular, the damage was concentration dependent, which agreed with the histopathology results. Meanwhile, the expression of mtDNA replication- and transcription-related genes was mostly significantly inhibited by thifluzamide at 1.90 and 2.85 mg/L. The functional state of mtDNA requires some factors for gene expression, DNA replication and DNA repair. These processes are eventually controlled by the cell nucleus because the requisite proteins are encoded by nuclear genes and imported into the mitochondria^25^. The ER plays a crucial role in many cellular processes, particularly in the folding and trafficking of proteins, carbohydrate and lipid metabolism, and detoxification^26^. Thus, nuclear fragmentation and ER fracture under TEM observation might result in faithful mtDNA maintenance, which would agree well with the expression inhibition of mtDNA replication- and transcription-related genes. mtDNA mutations cause specific mitochondrial diseases^27^,^28^. In vertebrates, mtDNA is present in multiple copies and is devoted to the expression of the respiratory chain complexes located on the inner mitochondrial membrane, which is important for oxidative phosphorylation and the production of cellular ATP^27^,^29^. This was confirmed by the expression inhibition of mitochondrial complex-related genes in the current study. It should be noted that in mitochondrial complex-related genes, the expression of *sdha* was significantly inhibited at even 0.19 mg/L. *sdha*, which encodes the major catalytic subunit of SDH, was more sensitive to thifluzamide^30^,^31^,^32^. The reduced expression level of *sdha* might suggest functional damage to SDH, which would agree well with the obviously low activity of SDH measured in this study. It is significant that SDH is located in the inner mitochondrial membrane and comprises a component of succinate dehydrogenase (complex II) in the electron transport chain (ECT)^33^,^34^. The activity of SDH parallels the number of mitochondria and is closely related to energy generation, which could reflect the function of mitochondria^35^. The activities of mitochondrial respiratory chain complexes are the most basic and most important indexes^36^ that reflect the mitochondrial function. The activity of SDH in this study was concentration dependent and obviously decreased at 2.85 mg/L, which might indicate that mitochondrial function was damaged. Thus, the decreased activity of SDH, the down-regulated levels of the related genes expression and the mitochondrial structure damage might lead to pathological changes and the developmental toxicity of embryos.

Oxidative stress is an important subject in aquatic toxicology^37^. Oxidative stress can result in oxidative damage and significant damage to cell structures^7^,^38^. Damage to mitochondrial structure and function increases ROS and causes oxidative stress^39^,^40^,^41^. In addition, SDH is closely related to the formation of ROS and has an antioxidant effect^42^,^43^. SDH mutations lead to oxidative stress, reduced lifespan, tumourigenesis and genomic instability^32^. A structural and functional analysis of SDH suggested a mechanism for ROS production at complex II during electron transport, exactly at the FAD sites of the sdha subunit^44^. Thus, the inhibition of SDH activity, the reduced transcription level of *sdha* and mitochondrial structure damage in the present study might have induced oxidative damage. Meanwhile, oxidative damage could disturb the copy of mtDNA and RNA and oxidate mitochondrial proteins and respiratory chain complexes, leading to abnormities in mitochondrial structure and function^45^. Oxidative damage and mitochondrial injury interact with each other in a circle. SOD, CAT and GPx could catalyse the transformation of H_2_O_2_ to oxygen and water to resist ROS accumulation^46^,^47^. The activities of SOD, CAT and GPx and the expression of the related genes could indicate the antioxidant capacity of the cell^48^,^49^. MDA is the product of lipid peroxide, and a change of its content reflects the degree of damage to cells attacked by the free radical^50^. The present study showed that thifluzamide could inhibit the activities of antioxidant enzymes, including SOD, CAT, and GPx, confirmed by the expression inhibition of genes encoding antioxidant enzymes in embryos after exposure. An obvious increase in the level of MDA in embryos was observed after exposure to 2.85 mg/L thifluzamide. Thus, it is possible that the decreased antioxidant enzyme activities (SOD, CAT, and GPx) and the expression of related genes resulted in ROS accumulation, ultimately leading to lipid peroxidation and mitochondrial damage. Similar results also showed a significant increase in MDA content accompanied by decreased SOD, CAT, and GPx activities in embryos exposed to difenoconazole (5.04 mg/L)^48^. These results suggest that thifluzamide provokes an oxidative damage. In addition, SOD could increase the rate of embryonic development, while a high ROS concentration decreased it^51^. The inhibition of antioxidant enzyme activities might have resulted in embryonic developmental delay in our study.

Apoptosis is an important toxic event^52^. Oxidative damage and mitochondrial injury could lead to cellular apoptosis^53^. Caspase-3, caspase-9 and apaf1 activation could lead to apoptosis^49^,^54^,^55^,^56^,^57^. Typically, Bcl-2 inhibits cell apoptosis^6^,^58^,^59^. As a pro-apoptotic member, Bax plays a key role in cell apoptosis^60^. The *bcl-2/bax* ratio is a determinant of susceptibility to apoptosis^61^. In the current study, the *bcl-2/bax* ratio had no obvious change at 2.85 mg/L. In contrast, mitochondrial membrane permeability changed due to mitochondrial membrane rupture, and the expression levels of *bax, apaf-1, Caspase-9* and *Caspase-3* were obviously up-regulated at 2.85 mg/L, which might result in cell apoptosis. These results were confirmed by TEM, which showed that cell apoptosis, including apoptosis body formation, occurred in the treatment groups. Moreover, caspase activities are the key markers of cellular apoptosis. Activated caspases can lead to cell apoptosis^62^. Caspase-3 activation can result in cellular apoptosis^63^. Caspase-9 activation allows for the activation of other caspases, which ultimately mediate the morphological and biochemical features of apoptosis^64^,^65^,^66^. In this study, the activity of Caspase-3 was obviously induced in all treatment groups, and that of Caspase-9 were significantly up-regulated at 1.90 and 2.85 mg/L, further confirming that cell apoptosis was induced. Similar results were also obtained in previous studies^54^,^66^,^67^. Therefore, we suggested that oxidative damage and mitochondrial injury could induce cell apoptosis, which might be responsible for the malformation and cell damage in the early life stages in the present study.

In addition, the immune system is an important indicator when assessing chemical toxicity. The basis of immunity is to eliminate foreign invaders. Some reports have shown that chemicals can interfere the immune system and exert immunotoxic on animals^68^,^69^. The adaptive immune system in zebrafish emerges from 4 to 6 weeks after fertilization, and only innate immunity exists in embryos^70^. Once the immune cells are activated, the expression of the immune genes is significantly up-regulated, leading to an immune response^71^. Chemokines, such as IL-8, ClC and CC-chem provide nonspecific immunity and are potential mediators of inflammation^72^,^73^. In the present study, immune-related gene expression was obviously up-regulated at 2.85 mg/L. The IL-8 content at all treatment groups was higher than that at control groups. These results indicated that the inflammatory response was induced after thifluzamide treatment, which might be interpreted as an adaptation during thifluzamide intoxication. Similar results also have been previously described^48^. However, cell apoptosis and oxidative damage could injure immune cells and immune function^74^,^75^. In particular, in the present study at 1.90 and 2.85 mg/L oxidative damage and apoptosis were obviously induced, which might lead to the decline of IL-8 content.

In addition, there are few references to the environmental fate of thifluzamide. Reports about the toxic effects of thifluzamide on aquatic life in paddy fields are limited. Thus, we aimed to explore the mechanism of toxic effects of thifluzamide on zebrafish embryos and to determine how best to use thifluzamide in the field while maintaining environmental safety.

Taking these results together, it could be hypothesized that mitochondrial structural damage and the activity inhibition of SDH are responsible for the developmental toxicity and malformation of embryos caused by thifluzamide, via inducing oxidative damage, apoptosis and immune response. The present study provides us with further insight into the toxicity mechanism of thifluzamide in embryos and suggests a mitochondrial focus for future research.

## Materials and Methods

### Chemicals and reagents

Standard water contained 2 mmol/L Ca^2+^, 0.5 mmol/L Mg^2+^, 0.75 mmol/L Na^+^ and 0.074 mmol/L K^+^ was used for the exposure experiments. Thifluzamide (95%) (CAS: 130000-40-7) was obtained from Beijing Huarong Biological Hormone Plant and the stock solution used for drug exposure was prepared with acetone AR and Tween-80. All of the other reagents utilized were analytical grade with the highest purity available.

### Embryo maintenance and exposure method

Wild type zebrafish were purchased from Beijing Hongdagaofeng Aquarium Department and maintained in flow-through feeding equipment (made by Esen Corp.) at 26°C with a photoperiod of 14/10 (light/dark)^76^. Mixed feed (market-finished bait, dried prawn, red worm) was fed to fish twice per day. The male parents were separated from females at least one week before spawning. We put fishes into spawning boxes overnight in a 2:1 male:female ratio, separated with isolation boards. New eggs born within one-half hour were collected and washed. 200 healthy embryos were placed in each beaker filled with test solutions (0.19, 1.90 and 2.85 mg/L) for exposure experiments. Test solutions were made up using standard water and were designed on the basis of pre-experiment data. Moreover, the blank control and solvent groups were used as controls. The solvent group contained the same acetone and Tween-80 contents with the highest dosage of solution. Each group ran in three replicates and lasted for 4 days. The exposure solution was renewed daily according to Mu *et al*.^76^. The external conditions during exposure experiments, including humidity, temperature and light cycle, were the same as the culture environment. The experiments were performed in accordance with current Chinese legislation and were approved by the Independent Animal Ethics Committee at the China Agricultural University.

### Histopathology

Five embryos for histological examination were taken from each group at the end of the exposure period and fixed in 10% formalin. The samples were dehydrated in a graded series of ethanol and embedded in glycol methacrylate. Step sections (4 mm thick) were prepared using a microtome (Ermainc AO820, Japan) and stained with haematoxylin and eosin. The sections were observed under light microscopy (Olympus BH-2, Japan)^4^.

### Subcellular structural analysis

At the end of the exposure period, 5 embryos in each group were selected for TEM (JEM-1230)^77^. Samples were fixed in 2.5% Glutaric dialdehyde (for at least 2 hours) and washed 3 times with 0.1 mL of phosphate buffer. The specimens were fixed in 1% osmic acid for 2 hours and washed with 0.1 mL of phosphate buffer for 3 times. Next, the specimens were dehydrated in a graded series of acetone and embedded in epoxy resin (SRURR). Ultrathin sections taken from selected areas were prepared using an ultramicrotome (LEICA UC6) and stained with uranyl acetate and lead citrate. Finally, the sections were examined using TEM (JEM-1230).

### Enzyme activity and MDA content analysis

At 4 days post-exposure (dpe), 50 embryos were randomly selected from each breaker for sample preparation, which was carried out as previously described^48^. The activities of SDH, SOD, CAT, GPx and the content of MDA were measured using assay kits (Jiancheng, Nanjing, China). The total protein content was determined using a bicinchoninic acid protein assay kit (CW Biotech, Beijing, China).

### Caspase activity measurement and IL-8 content analysis

Thirty embryos from each breaker were selected to measure the Caspase-3 and Caspase-9 activities. The enzyme activities were determined using assay kits (Beyotime Institute of Biotechnology, Haimen, China). Thirty embryos were selected to measure the levels of IL-8 using fish IL-8 enzyme-linked immunosorbent assay (ELISA) kits (Jiancheng, Nanjing, China) with a microtitre plate reader at 450 nm (Multiskan MK3).

### Gene expression analysis

At the end of the exposure period, 30 embryos were selected for total RNA^48^. The transcription of genes related to oxidation, apoptosis, immunity and mitochondria was measured by quantitative real-time polymerase chain reaction (PCR) according to Mu *et al*.^48^. The primers are listed in Table 3. The transcripts were quantified using the 2^−ΔΔCt^ method.

### Water analysis

Water analysis was performed according to Yang *et al*.^4^. Each replicate for each treatment was analysed twice at the beginning of the exposure period and 24 h post-exposure (hpe) before solution renewal.

### Data analysis

The statistical analyses were undertaken using SPSS 16.0 (SPSS, Chicago, IL, USA). Differences between control group and treated groups were determined by a one-way analysis of variance (ANOVA), with Dunnett post hoc comparison; *P* < *0.05* was considered statistically significant and was corrected for multiple comparisons using Bonferroni correction.

## Additional Information

**How to cite this article**: Yang, Y. *et al*. Biological response of zebrafish embryos after short-term exposure to thifluzamide. *Sci. Rep.* **6**, 38485; doi: 10.1038/srep38485 (2016).

**Publisher’s note:** Springer Nature remains neutral with regard to jurisdictional claims in published maps and institutional affiliations.

## Figures and Tables

**Table 1:** Chemical analysis results.

Test stage	Treatment (mg/L)	Content at 0 hpe (mg/L)	Deviation (%)	Content at 24 hpe (mg/L)	Deviation (%)
Embryo	control	n.d.^a^		n.d.	
	0.19	0.163 ± 0.009	−14.21^b^	0.157 ± 0.006	−17.37
	1.90	2.019 ± 0.029	6.27	1.985 ± 0.017	4.47
	2.85	2.657 ± 0.037	−6.77	2.580 ± 0.087	−9.47

^a^n.d. = not detected.

^b^deviation = (actual content-nominal content)/nominal content.

**Table 2:** Microscopic Lesions of Tissues of Zebrafish embryos/larvae Exposed as Fertilized Eggs to Graded Doses of Thifluzamide.

hpe^a^	Treatment (mg/L)	Head damage	Yolk sac edema	Pericardial edema	Uninflated gas bladder
96	control	0/15	0/15	0/15	0/15
	0.19	0/15	4/15	0/15	0/15
	1.90	3/15	6/15	7/15	5/15
	2.85	8/15	11/15	11/15	10/15

^a^Hours post-exposure.

**Table 3:** Sequences of the primer pairs used in the real-time quantitative PCR reactions.

Gene	Forward primer	Reverse primer	References
*β-actin*	CGAGCAGGAGATGGGAACC	CAACGGAAACGCTCATTGC	78
*BAX*	GGCTATTTCAACCAGGGTTCC	TGCGAATCACCAATGCTGT	78
*BCL2*	AGGAAAATGGAGGTTGGGATG	TGTTAGGTATGAAAACGGGTGGA	78
*Caspase 3*	CCGCTGCCCATCACTA	ATCCTTTCACGACCATCT	78
*p53*	GGGCAATCAGCGAGCAAA	ACTGACCTTCCTGAGTCTCCA	78
*APAF1*	TTCTACAGTAAACGCCCACC	TATCTAGTATTTCCCCATATTCC	78
*Caspase9*	AAATACATAGCAAGGCAACC	CACAGGGAATCAAGAAAGG	78
*IFN*	TGCGTCTACTTGCGAATGGCTTGG	ACCTGGTCCTCCACCTTTGACTTGT	79
*IL-8*	GTCGCTGCATTGAAACAGAA	CTTAACCCATGGAGCAGAGG	79
*C1C*	CATCCGGCCAGCTCTGCTTGAAT	CCACTCTTGACCTCCTGTGCTCTCT	79
*CC-chem*	CTTTGACGCATGGAGGATTT	TGCAGCTCAACCAGAAGATG	79
*Mn–Sod*	CCGGACTATGTTAAGGCCATCT	ACACTCGGTTGCTCTCTTTTCTCT	79
*Cu/Zn–Sod*	GTCGTCTGGCTTGTGGAGTG	TGTCAGCGGGCTAGTGCTT	79
*Cat*	AGGGCAACTGGGATCTTACA	TTTATGGGACCAGACCTTGG	79
*GPx*	AGATGTCATTCCTGCACACG	AAGGAGAAGCTTCCTCAGCC	79
*18rs rrn*	AGCGTGCGGGAAACCACGAG	AAGCCGCAGGCTCCACTCCT	80
*polg1*	GGTGACCAGTGAAGACCGATA	GTCCACTGCGCTAAAGAAGG	80
*tk2*	CCTGTATGAGGACTGGCTGA	TCTGTTCTCCTCAAACTGATGC	80
*twinkle*	TGTGGGCTGACAAGTTTGAGG	TGTCCACAGACAGATTTTCTTG	80
*polrmt*	ACCCGCTGCCGCCTTATTTT	TCCAGCGAGCTCTGCTTCTTC	80
*tfam*	GCGAAAGATTGCCCAGCAGT	TTGTCGTTTTTCCTCCGCAAA	80
*mt-nd1*	AGCCTACGCCGTACCAGTATT	GTTTCACGCCATCAGCTACTG	80
*ndufs4*	TGTAGGCTGGCAGAGGGACA	GACAGGCCGAAACAGGATGG	80
*Shda*	TGGTATGCCGTTCAGCCGTA	GGCCAAGTCTTTGGCATTGG	80
*uqcrc2*	GACCTCACGGGAAGGGTGAA	TCAGTGTGCTGGTGCTGCTG	80
*cox5ab*	GGTCACCGGAGCTTCAGGAT	TCGAGCCGAGAGGTAGAAAAACC	80
*atp5α1*	TTCTTGGAGCCGACACTGGA	CGAACACCACAACACCAACG	80

All of the sequences are shown 5′→3′.
